# Tailoring Analgesia in Oesophagectomy: One Size Does Not Fit All

**DOI:** 10.12669/pjms.42.9.16314

**Published:** 2026-09

**Authors:** Faraz Mansoor, Zubair Shabbir Khanzada, Zala Saleem

**Affiliations:** 1 Faraz Mansoor, MBBS, FCPS (Anaesthesia), FCPS (Critical Care Medicine). International Fellowship in Critical Care Medicine (UK), Consultant Anaesthetist, Department of Anaesthesia, Shaukat Khanum Memorial Cancer Hospital and Research Centre, Peshawar, Pakistan; 2 Zubair Shabbir Khanzada, MBBS, MRCS, MSc, MD, PhD, FRCS, CCT (UK). Consultant Surgical Oncologist and Foregut Surgeon, Shaukat Khanum Memorial Cancer Hospital and Research Centre, Peshawar, Pakistan; 3 Zala Saleem, MBBS. Resident Doctor, Department of Anesthesia, Shaukat Khanum Memorial Cancer Hospital and Research Centre, Peshawar, Pakistan

There is absolutely no doubt that oesophagectomy is one of the most complex procedures in cancer surgery. It is also true that in some cases a thoracic epidural analgesia (TEA) might not work, and in some cases it can lead to complications such as a dural tap and hypotension, for example. Over the last few decades, we have observed the evolution of minimally invasive surgery techniques. In addition, the introduction of enhanced recovery after surgery (ERAS) pathways has pushed healthcare professionals to opt for less invasive options owing to their inherent advantages of quick recovery and cost-effectiveness. Moreover, the availability of alternative and less invasive analgesic options has led some people to argue that the routine use of TEA is not required in patients undergoing oesophagectomy. However, TEA is still recommended in the ERAS guidelines and the EUPEMEN oesophagectomy protocol.^1^,^2^

A detailed review about the historical background of the TEA is beyond the scope of this discussion. There is good quality evidence that it provides several advantages, including reduced postoperative respiratory complications, a shorter hospital stay, and a decreased risk of anastomosis leakage. In addition, it offers superior pain control compared with systemic analgesia, facilitating early mobilization and improved pulmonary function. These benefits contribute to enhanced postoperative recovery and overall patient outcomes, particularly in major thoracic and upper abdominal surgeries.^3^ Nevertheless, limitations and contraindications associated with thoracic epidural analgesia have prompted the investigation of alternative analgesic strategies for patients undergoing oesophagectomy.

Several alternative regional analgesic techniques, including paravertebral block, erector spinae plane block, intercostal nerve block, transverse abdominis plane block, and serratus anterior plane block, have been explored to improve postoperative analgesia in patients undergoing oesophagectomy. However, paravertebral block is not superior to the TEA in functional recovery which was one of the main finding in the PEPMEN randomized controlled trial.^4^ In addition to this the same trial reported that paravertebral block patients reported higher pain scores and lower quality of recovery compared with TEA on postoperative day one and two. It is important to note that in Pakistan, relatively few patients require vasopressor support solely due to the placement of thoracic epidural analgesia, despite hypotension frequently being cited as one of the major disadvantage of thoracic epidural analgesia. Moreover, guidelines indicate that the bleeding risk associated with thoracic epidural analgesia and paravertebral block is comparable; therefore, the same anticoagulation precautions must be observed. Consequently, paravertebral block cannot be regarded as a safer alternative to TEA in patients at increased risk of bleeding.^5^ In terms of the erector spinae block, there is insufficient evidence to consider it the definitive first-choice regional block; however it may be used in cases of a failed epidural as part of the multimodal analgesia regimen.^6^ No conclusion can be drawn from the studies regarding the serratus anterior, transversus abdominis plane block, or intercostal nerve block, as these were compared with patient-controlled analgesia rather than thoracic epidural.^7^

To sum up, oesophagectomy is a highly complex procedure, and effective postoperative pain management is essential for optimal recovery. In planning analgesia, all relevant factors must be considered, including the ERAS pathways’ recommendation for multimodal techniques, the type of the surgical approach; minimally invasive or open thoracotomy, two or three-stage, underlying comorbidities such as COPD or interstitial lung disease, and tumor location. Tailoring the analgesic strategy to these variables ensures the best outcomes for individual patient. Therefore, one size does not fit all, and while thoracic epidural analgesia remains the gold standard, alternative regional techniques should be reserved for specific circumstances, emphasizing a personalized, procedure specific approach to optimize recovery and patient safety.

## Author Contributions:

**FM** conceived the idea for the manuscript and prepared the initial draft.

**ZSK** critically reviewed the manuscript and approved the final version.

**ZS** did reference management.
